# Baseline atherogenic index of plasma and its trajectory predict onset of type 2 diabetes in a health screened adult population: a large longitudinal study

**DOI:** 10.1186/s12933-025-02619-6

**Published:** 2025-02-07

**Authors:** Yongbing Sun, Xinbei Lin, Zhi Zou, Caiwen Zhao, Ao Liu, Jing Zhou, Zhonglin Li, Xiaoling Wu, Shewei Dou, Jiadong Zhu, Tao Li, Xue Lv, Yong Wang, Yongli Li

**Affiliations:** 1 Department of Medical Imaging, Zhengzhou University People’s Hospital, Henan Provincial People’s Hospital, # 7 Wei Wu Road, Zhengzhou, 450003 Henan China; 2 The Third Affiliated Hospital of Zhengzhou University, #7 Kungfu Street, Zhengzhou, 450052 Henan China; 3 Henan Provincial Research Center of Clinical Medicine of Nephropathy, Henan Provincial People’s Hospital, Zhengzhou University People’s Hospital, Henan University People’s Hospital, Zhengzhou, 45003 China; 4 Department of Nuclear Medicine, Henan Provincial People’s Hospital, Zhengzhou, 450003 Henan China; 5 Department of Health Management, Chronic Health Management Laboratory, Henan Provincial People’s Hospital, Zhengzhou University People’s Hospital, Zhengzhou, 450003 Henan China; 6 Fuwaihua Central Vascular Disease Hospital, #1 Fuwai Avenue, Zhengzhou, 451464 Henan China; 7 Henan Provincial People’s Hospital, #7 Wei Wu Road, Zhengzhou, 450003 Henan China

**Keywords:** Plasma atherogenic index, Type 2 diabetes mellitus, Trajectory analysis, Prospective longitudinal study

## Abstract

**Background:**

The Atherogenic Index of Plasma (AIP) is a novel biomarker for assessing the severity of atherosclerosis and has been shown to be closely associated with the risk of Type 2 Diabetes Mellitus (T2DM). However, no prospective cohort study has comprehensively evaluated both the immediate risk stratification through baseline AIP and the long-term risk assessment through multi-time point AIP trajectories in health screened adults in relation to T2DM risk.

**Methods:**

This longitudinal study included data from 42,850 participants who underwent health check-ups at Henan Provincial People’s Hospital between January 2018 and August 2024. AIP was calculated as the logarithm of the ratio of triglycerides (TG) to high-density lipoprotein cholesterol (HDL-C). The Kaplan-Meier method was employed to analyze the incidence of T2DM across different AIP groups. A Cox model with restricted cubic splines assessed the dose-response relationship between AIP and T2DM risk, while latent class trajectory models (LCTM) evaluated the trends of AIP over multiple time points. Cox proportional hazards models were used to examine the relationship between baseline AIP quartiles, AIP trajectories, and T2DM risk.

**Results:**

During an average follow-up of 47.95 months, 3,228 participants developed T2DM. Stratifying by baseline AIP quartiles revealed that higher AIP levels were associated with an increased risk of T2DM. Compared to the lowest quartile, the highest quartile had a hazard ratio (HR) of 2.10 (95% CI: 1.74, 2.53). The LCTM identified three trajectory patterns for AIP: with the low-stable group as the reference, the medium-stable and high-stable groups had HRs of 1.72 (95% CI: 1.50, 1.96) and 2.50 (95% CI: 2.06, 3.03), respectively, indicating a significantly elevated risk of T2DM (*P* < 0.05).

**Conclusion:**

Elevated baseline AIP levels, medium stable trajectories and high stable trajectories are associated with an increased risk of T2DM in health screened adults.

**Supplementary Information:**

The online version contains supplementary material available at 10.1186/s12933-025-02619-6.

## Introduction

Type 2 Diabetes Mellitus (T2DM) is a complex and chronic metabolic disorder that poses a significant global public health challenge [1]. Recent estimates suggest that around 529 million people globally are living with T2DM, with projections indicating this figure could rise to 1.31 billion by 2050 [2]. Presently, China accounts for approximately 140 million individuals with T2DM, the highest prevalence worldwide [3], thereby imposing a considerable burden on global healthcare systems. The pathogenesis of T2DM involves complex interactions between metabolic disturbances, with lipid metabolism playing a crucial role through multiple mechanisms [4]. Specifically, lipotoxicity impairs β-cell function through oxidative stress [5], while elevated free fatty acids induce insulin resistance by disrupting insulin signaling pathways and triggering chronic inflammation [6, 7]. These pathophysiological processes manifest clinically as increased triglycerides (TG) and decreased high-density lipoprotein cholesterol (HDL-C) [8], suggesting that monitoring lipid profiles may be crucial for T2DM prevention [9].

The Atherogenic Index of Plasma (AIP), calculated as the logarithm of the TG to HDL-C ratio [10, 11], offers distinct advantages over conventional lipid parameters by better reflecting lipoprotein particle size and atherogenicity [12]. Studies have demonstrated AIP’s strong correlation with cardiovascular risk [13, 14] and T2DM development [15, 16]. Recent analyses have established AIP as an independent predictor of incident diabetes, outperforming traditional lipid ratios in risk assessment [17]. A multicenter longitudinal study revealed that maintaining an AIP below 0.04 is crucial for normal glucose homeostasis [18], while cross-sectional research has confirmed strong associations between elevated AIP and T2DM risk [16]. However, most existing studies have relied on single-point measurements, overlooking the potential significance of temporal AIP changes in disease progression [19]. Furthermore, previous studies have typically focused on either baseline AIP levels or trajectory patterns in isolation, limiting our comprehensive understanding of AIP’s role in T2DM development. An integrated evaluation incorporating both immediate risk stratification through baseline AIP and long-term risk assessment through trajectory patterns would provide more complete insights into the relationship between AIP and T2DM risk.

To address this knowledge gap, we conducted a longitudinal study using health check-up data from Henan Provincial People’s Hospital. Using latent class trajectory modeling (LCTM), we investigated both baseline AIP levels and their temporal changes in relation to T2DM risk. This research aims to enhance our understanding of AIP’s predictive value and its potential application in T2DM prevention strategies.

## Materials and methods

### Study design and population

**Fig. 1:**
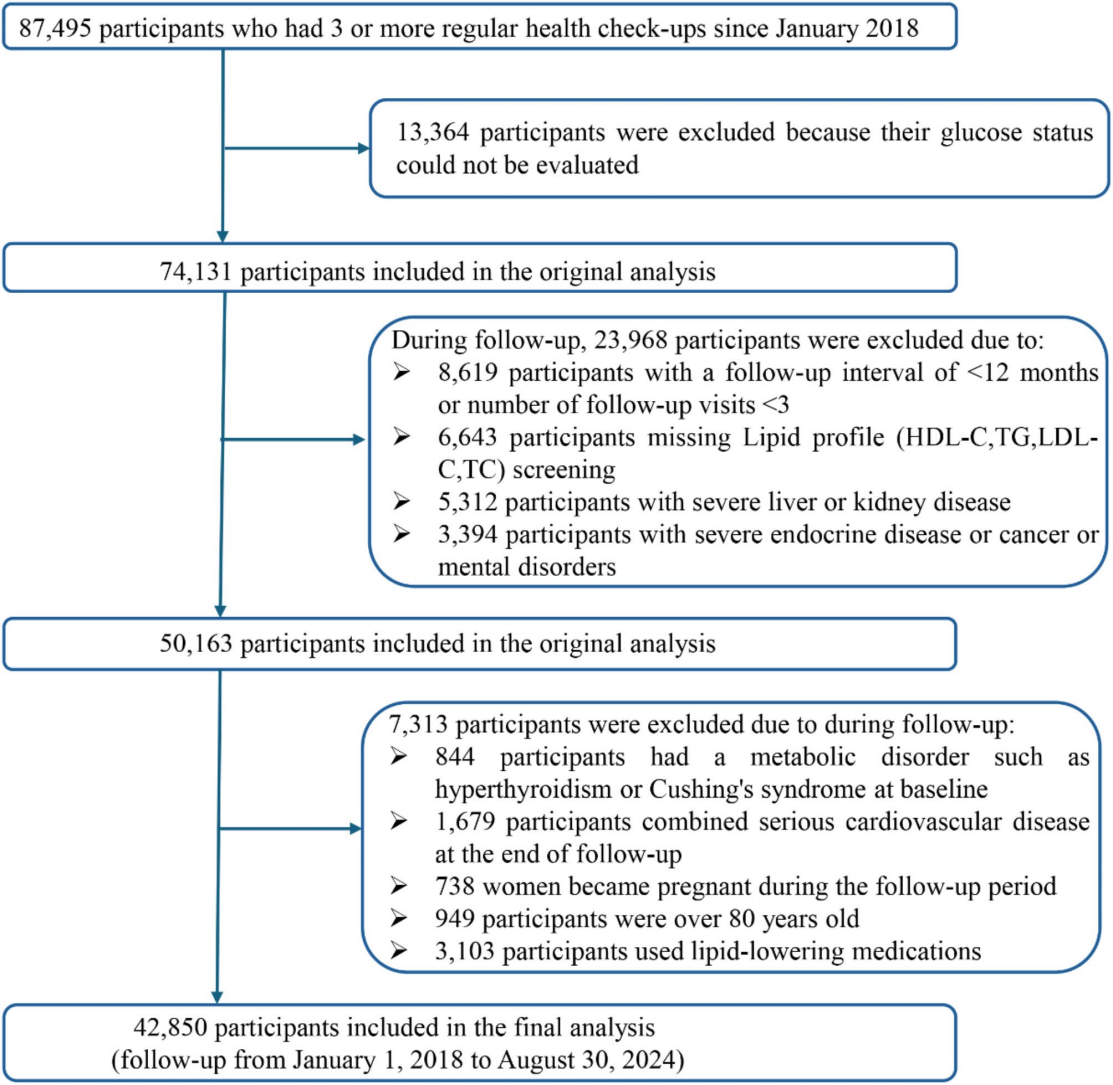
Participant screening flowchart

This retrospective cohort study used real-world data collected from adults aged 20–80 who received regular check-ups at the Health Management Center of Henan Provincial People’s Hospital between January 2018 and August 2024. All participants were registered members of the center (employees or their parents) with employer-sponsored annual examinations, ensuring a long-term relationship with the center. Medical check-ups occurred every 6 to 12 months, and each participant had at least three visits. Follow-up began at the first examination. Participants diagnosed with T2DM during follow-up were censored at diagnosis, whereas those who remained free of T2DM were followed until August 31, 2024. During the initial screening, 13,364 participants were excluded because their glucose status could not be evaluated. In addition, 31,281 participants were excluded due to incomplete lipid data, severe cardiovascular conditions requiring hospitalization (heart failure NYHA III–IV, unstable angina, or myocardial infarction), marked liver impairment (liver enzymes exceeding three times the upper limit), reduced kidney function (eGFR < 60 mL/min/1.73 m^2^), endocrine disorders (Hyperthyroidism or hypothyroidism (TSH < 0.27 or > 4.2 mIU/L), pituitary disorders, adrenal disorders, or any other endocrine conditions that may affect glucose and lipid metabolism.) that could affect glucose or lipid metabolism, cancer or mental health conditions, pregnancy, age ≥ 80 years at the end of follow-up, or use of lipid-lowering medications during follow-up. In total, 42,850 participants who were free of T2DM at baseline and had complete medical records throughout follow-up were included in the final analysis. A summary of the selection process is shown in Fig. 1.

### Laboratory measurements

All researchers received standardized training to ensure objectivity and accuracy. Each participant’s unique identification number was used to match health examination records, medical history, and questionnaire responses. All data collection procedures followed established standard operating protocols. Before each examination, researchers used a structured questionnaire to gather demographic information, including histories of endocrine disorders, liver or kidney disease, cancer, and statin use. Upon completion of the questionnaire, a two-person verification team organized, compiled, and validated the collected data. The database was regularly maintained and audited for quality. Any errors or missing information were confirmed directly with participants or verified by telephone.

All participants provided fasting venous blood samples at 8:00 a.m. after a 12-hour overnight fast. Most biochemical parameters including total protein (TP), alanine aminotransferase (ALT), aspartate aminotransferase (AST), blood urea nitrogen (BUN), creatinine (Cre), uric acid (UA), fasting blood glucose (FBG), total cholesterol (TC), low-density lipoprotein cholesterol (LDL-C), TG, and HDL-C were measured using an Olympus AU 5400 automatic biochemical analyzer (Olympus, Shizuoka, Japan). HbA1c was measured by high-performance liquid chromatography using the Pumon H9 glycated hemoglobin analyzer (Pumon, Shanghai, China). All measurements were performed according to standard laboratory protocols.

### Assessment of AIP

The AIP was calculated using the formula log10(TG/HDL-C) [11]. To provide comprehensive risk assessment, we employed two complementary analytical approaches: quartile analysis of baseline AIP for immediate risk stratification, and trajectory analysis for long-term risk evaluation. Baseline AIP was divided into quartiles to assess the initial risk distribution, while trajectory analysis was performed to capture the dynamic changes in AIP over time. Baseline AIP was derived from each participant’s initial TG and HDL-C measurements. The average AIP was determined by calculating individual AIP values at each follow-up visit from 2018 to 2024 and then taking the arithmetic mean of these values. A LCTM was used to categorize participants into three groups based on changes in their AIP levels.

### Outcome

The primary endpoint was the onset of T2DM, and the secondary endpoint was the study completion date (August 31, 2024). Type 2 diabetes was diagnosed according to the American Diabetes Association criteria [20]. Diagnosis was confirmed when either of the following conditions was met: (1) prior clinical diagnosis with documented use of glucose-lowering medications, or (2) in the absence of unequivocal hyperglycemia, diagnosis requires two abnormal test results from either the same test repeated on a different day or two different tests at the same visit (FBG ≥ 7.0 mmol/L, HbA1c ≥ 6.5%, or 2-hour FBG ≥ 11.1 mmol/L during OGTT). For participants with a single abnormal result, a second confirmatory test was performed within 2–4 weeks. When discordant results occurred, the abnormal test was repeated for confirmation. All diagnoses were verified through our hospital’s electronic medical record system. All participant diagnoses were verified by cross-referencing medical records in the hospital’s electronic record system.

### Definitions of variables

BMI was calculated as weight (kg) / height^2^ (m^2^).

Hypertension was defined as systolic blood pressure (SBP) ≥ 140 mmHg or diastolic blood pressure (DBP) 90 ≥ mmHg on two consecutive measurements, self-reported hypertension, taking antihypertensive medication, or undergoing antihypertensive treatment [21].

Current smoking was defined as self-reported smoking by the participants. Current drinking was defined as consuming at least one alcoholic beverage per week in the 12 months before the health examination.

Estimated glomerular filtration rate (eGFR) was calculated using the formula: eGFR = 175 * serum creatinine^− 1.154^ * age^− 0.203^ * 0.742 (if female) * 1.212 (if black) [22]. eGFR is expressed in mL/min/1.73 m^2^, with serum creatinine in mg/dL and age in years.

### Statistical analysis

All continuous variables were tested for normality. Normally distributed variables were reported as mean ± standard deviation, whereas skewed variables were presented as median (interquartile range). Between-group differences were evaluated using the t-test or rank-sum test. Categorical variables were expressed as frequencies and percentages, and comparisons were made using the chi-square test.

Baseline AIP was divided into quartiles (Q1, Q2, Q3, and Q4), with Q1 indicating the lowest value and Q4 the highest. Follow-up time was calculated from baseline to the last visit, encompassing the final recorded biochemical data and T2DM events. Kaplan–Meier curves were plotted for each AIP group, and differences were tested by the log-rank method.

A LCTM was applied to examine changes in AIP over multiple time points [23]. The optimal number of trajectory groups was determined using the Bayesian information criterion. Model fit was evaluated by: (1) calculating the mean posterior probability of group membership (≥ 0.7 was considered acceptable), (2) assessing correct classification likelihood, and (3) comparing the estimated group probabilities with observed group proportions. Parameters were estimated through the expectation–maximization algorithm.

Cox proportional hazards models (univariable and multivariable) were used to assess the relationship between AIP and T2DM risk. Three models were constructed: an unadjusted model; Model I adjusted for demographic factors (sex, age, ethnicity, and marital status); and Model II fully adjusted for all potential confounders. Covariates were selected by excluding those with a variance inflation factor (VIF) > 10. A restricted cubic spline Cox model was employed to explore the dose–response relationship between AIP and T2DM. To assess the robustness of the findings, sensitivity analyses were performed by (1) incorporating mean AIP into the fully adjusted Cox model, and (2) testing various AIP cutoff points. To facilitate comparison with trajectory groups and validate our main findings, we also conducted sensitivity analyses by categorizing baseline AIP into tertiles. The baseline characteristics across AIP tertiles and their associations with T2DM risk were analyzed using the same statistical approaches as the quartile analyses. To compare the predictive capabilities of baseline AIP categorization and trajectory patterns for T2DM risk, we calculated incidence rates and hazard ratios for both classification methods.

All statistical analyses were conducted using R version 4.4.1 (R Foundation) and EmpowerStats (http://www.empowerstats.com, X&Y Solutions, Inc., Boston, MA). All tests were two-sided, and a *P*-value < 0.05 was considered statistically significant.

### Ethics declarations

This retrospective longitudinal study was conducted in compliance with the Declaration of Helsinki and received approval from the Ethics Committee of Henan Provincial People’s Hospital (Ethics No: 2021 Lunar Review No. 68). Informed consent was obtained from all participants, and their identifying information was re-coded to maintain the anonymity of personal data used in the study.

## Results

### Baseline characteristics by baseline AIP quartiles

To comprehensively evaluate the relationship between AIP and T2DM risk, we first analyzed the association between baseline AIP levels and T2DM incidence. A total of 42,850 adult participants undergoing health examinations were included, with an average age of 44.87 ± 14.81 years; among them, 21,728 (50.71%) were male. The mean baseline AIP level for the entire cohort was 0.01, and participant characteristics are summarized in Supplementary Table S1. Participants were stratified into quartiles according to their baseline AIP levels. Compared with other groups, participants in the highest quartile (Q4) were older, had a higher proportion of males, higher BMI, and a greater percentage of married individuals, as well as higher rates of alcohol use, smoking, and hypertension. In addition, participants in Q4 showed significantly higher levels of TP, ALT, AST, BUN, Cre, UA, FBG, HbA1c, TC, LDL-C, and TG, while eGFR rate and HDL-C levels were lower (all *P* < 0.001). Q4 also had the highest incidence of T2DM (12.98%). These findings are summarized in Table 1.

**Table 1:** Baseline characteristics according to the baseline AIP index quartiles

Characteristics	Q1 (-0.98, -0.19)	Q2 (-0.19, -0.01)	Q3 (-0.01, 0.19)	Q4 (0.19, 1.55)	*P*-value
N	10,711	10,712	10,711	10,716	
AIP	-0.31 (-0.39, -0.25)	-0.10 (-0.15, -0.06)	0.08 (0.03, 0.13)	0.34 (0.26, 0.47)	< 0.001
Mean AIP	-0.26 (-0.34, -0.18)	-0.08 (-0.15, 0.00)	0.09 (0.01, 0.16)	0.31 (0.21, 0.43)	< 0.001
Age, years	41.23 ± 14.42	45.90 ± 15.05	47.08 ± 14.52	48.78 ± 13.47	< 0.001
Sex, n (%)					< 0.001
Female	7,902 (73.77)	5,812 (54.26)	4,358 (40.69)	3,050 (28.46)	
Male	2,809 (26.23)	4,900 (45.74)	6,353 (59.31)	7,666 (71.54)	
BMI, kg/m^2^	21.95 ± 2.72	23.63 ± 2.98	24.94 ± 3.01	26.21 ± 3.02	< 0.001
Ethnic group, n (%)					0.402
Non-han	216 (2.02)	187 (1.75)	176 (1.64)	190 (1.77)	
Han	10,495 (97.98)	10,525 (98.25)	10,535 (98.36)	10,526 (98.23)	
Marriage status, n (%)					< 0.001
Unmarried	2,253 (21.03)	1,312 (12.47)	899 (8.57)	610 (5.85)	
Married	8,458 (78.97)	9,213 (87.53)	9,587 (91.43)	9,811 (94.15)	
Current drinking, n (%)					< 0.001
No	10,678 (97.82)	9,947 (92.86)	9,637 (89.97)	8,601 (80.26)	
Yes	233 (2.12)	765 (7.14)	1,074 (10.03)	2,115 (19.74)	
Current smoking, n (%)					< 0.001
No	10,478 (97.10)	9,778 (91.28)	9,256 (86.42)	8,536 (79.66)	
Yes	311 (2.90)	934 (8.72)	1,455 (13.58)	2,180 (20.34)	
Hypertension, n (%)					< 0.001
No	9,515 (88.83)	8,712 (81.33)	8,016 (74.84)	7,505 (70.04)	
Yes	1,196 (11.17)	2,000 (18.67)	2,695 (25.16)	3,211 (29.96)	
TP, g/L	71.81 ± 4.00	71.92 ± 4.05	72.20 ± 4.18	72.68 ± 4.12	< 0.001
ALT, U/L	14.10 (11.00–19.00)	16.70(12.60–22.90)	19.70(14.78–27.30)	24.00(17.60–34.90)	< 0.001
AST, U/L	20.11 ± 10.00	21.06 ± 8.95	22.11 ± 8.66	24.10 ± 13.42	< 0.001
BUN, mmol/L	4.80 ± 1.29	4.90 ± 1.27	5.00 ± 1.28	5.07 ± 1.21	< 0.001
Cre, µmol/L	57.53 ± 13.75	61.32 ± 13.84	64.45 ± 19.17	66.85 ± 14.04	< 0.001
UA,µmol/L	272.75 ± 68.05	303.82 ± 76.73	332.24 ± 82.81	368.75 ± 88.43	< 0.001
eGFR, mL/min/1.73m^2^	114.26 ± 21.12	110.44 ± 21.16	106.65 ± 20.64	107.36 ± 19.74	< 0.001
FBG, mmol/L	4.58 ± 0.43	4.70 ± 0.47	4.79 ± 0.51	4.90 ± 0.54	< 0.001
HbA1c, (%)	5.47 ± 0.38	5.54 ± 0.38	5.61 ± 0.37	5.63 ± 0.38	< 0.001
TC,mmol/L	4.59 ± 0.83	4.76 ± 0.88	4.93 ± 0.92	5.10 ± 0.95	< 0.001
LDL-C, mmol/L	2.39 ± 0.64	2.70 ± 0.70	2.86 ± 0.74	2.90 ± 0.74	< 0.001
TG, mmol/L	0.79 ± 0.17	1.14 ± 0.21	1.58 ± 0.29	2.89 ± 1.49	< 0.001
HDL-C, mmol/L	1.65 ± 0.28	1.43 ± 0.23	1.28 ± 0.20	1.10 ± 0.19	< 0.001
T2DM, n (%)					< 0.001
No	10,409 (97.18)	10,106 (94.34)	9,809 (91.58)	9,419 (87.02)	
Yes	302 (2.82)	606 (5.66)	902 (8.42)	1,391 (12.98)	

AIP, plasma atherogenic index; BMI, body mass index; TP, total protein; ALT, alanine aminotransferase; AST, aspartate transaminase; BUN, blood urea nitrogen; Cre, Creatinine; UA, Uric acid; eGFR, estimated glomerular filtration rate; FBG, fasting blood glucose; HbA1c, Glycosylated hemoglobin; TC, total cholesterol; LDL-C, low-density lipoprotein cholesterol; TG, triglycerides; HDL-C, high-density lipoprotein cholesterol; T2DM, type 2 diabetes mellitus. Except for the ALT, AIP, and mean AIP which is expressed as medians (upper and lower quartiles), all other variables are expressed as mean ± standard deviation or counts (percentages)

### Association between baseline AIP levels and the timing of T2DM onset

**Fig. 2:**
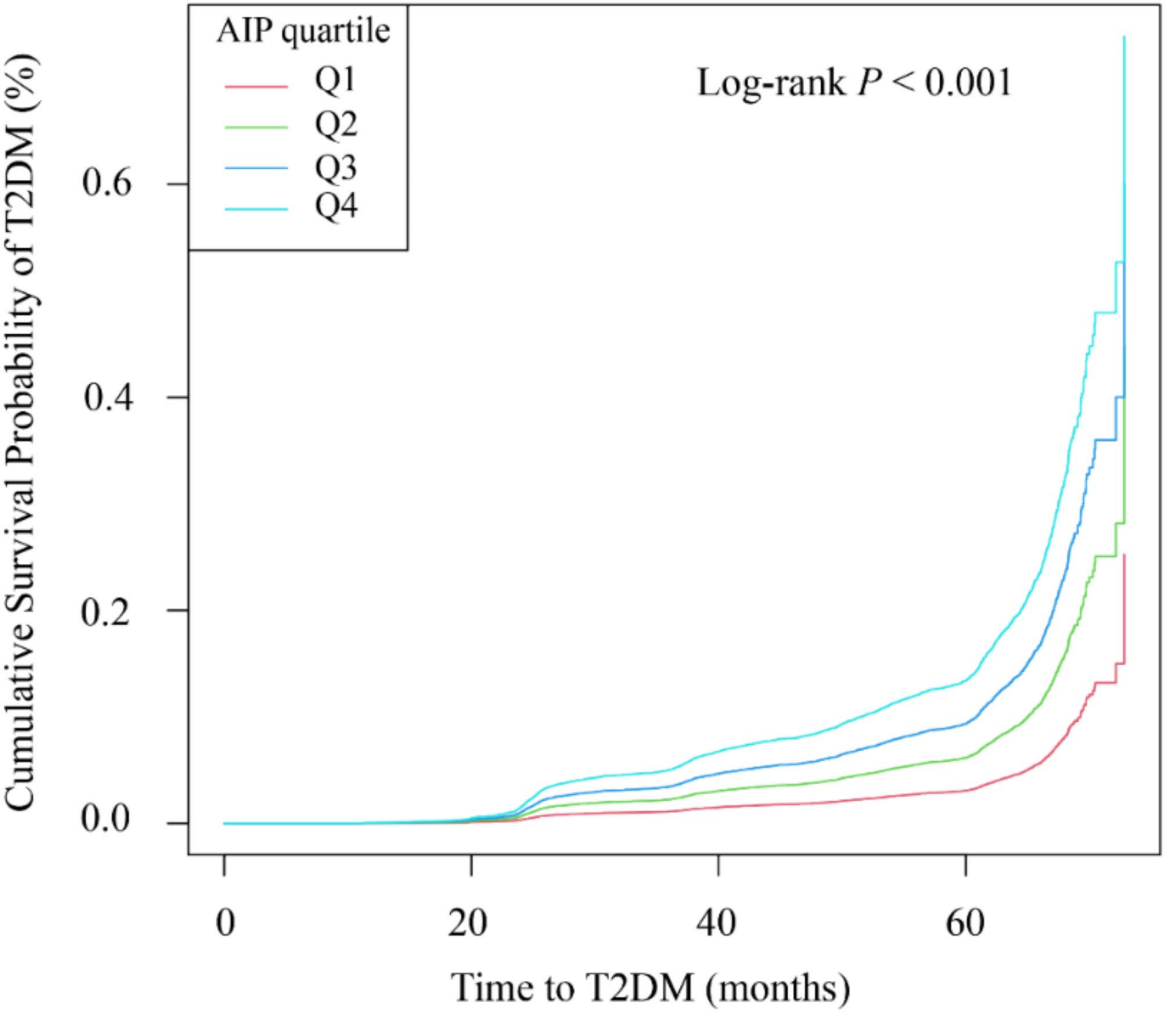
Kaplan-Meier survival analysis curves and baseline AIP quartiles for T2DM disease. y-axis represents cumulative survival, and x-axis represents duration of follow-up. AIP, plasma atherogenic index; T2DM, type 2 diabetes mellitus

The study enrolled 42,850 participants with an average follow-up of 47.95 months (median: 50.43; interquartile range: 36.60–61.03; range: 16.77–71.80). During this period, 3,201 adults developed T2DM. The Kaplan–Meier survival curves showed significant differences in T2DM incidence across the AIP quartiles, indicating that higher AIP was associated with an increased incidence of T2DM (log-rank *P* < 0.001), as illustrated in Fig. 2. To examine the robustness of our findings and address the population distribution differences between baseline AIP groups and trajectory patterns, we performed sensitivity analyses by dividing participants into AIP tertiles (low, medium, high). The baseline characteristics across AIP tertiles (Supplementary **Table S2**) showed similar patterns to those observed in quartile analyses. The Kaplan-Meier analysis of these three groups demonstrated significant differences in T2DM incidence (log-rank *P* < 0.001), with the high AIP tertile showing the greatest cumulative incidence, followed by the medium and low tertiles (Figure S1). The incidence rates of T2DM were 3.40%, 6.89%, and 11.46% for baseline AIP tertiles (T1-T3), respectively. After adjusting for potential confounders in Cox proportional hazards regression analysis, compared with the lowest tertile, the hazard ratios for T2DM were 1.30 (95% CI: 1.21–1.51) for T2 and 1.72 (95% CI: 1.48–1.99) for T3 (Supplementary Table S3). These results from the tertile analysis consistently supported our main findings from the quartile analysis.

**Fig. 3:**
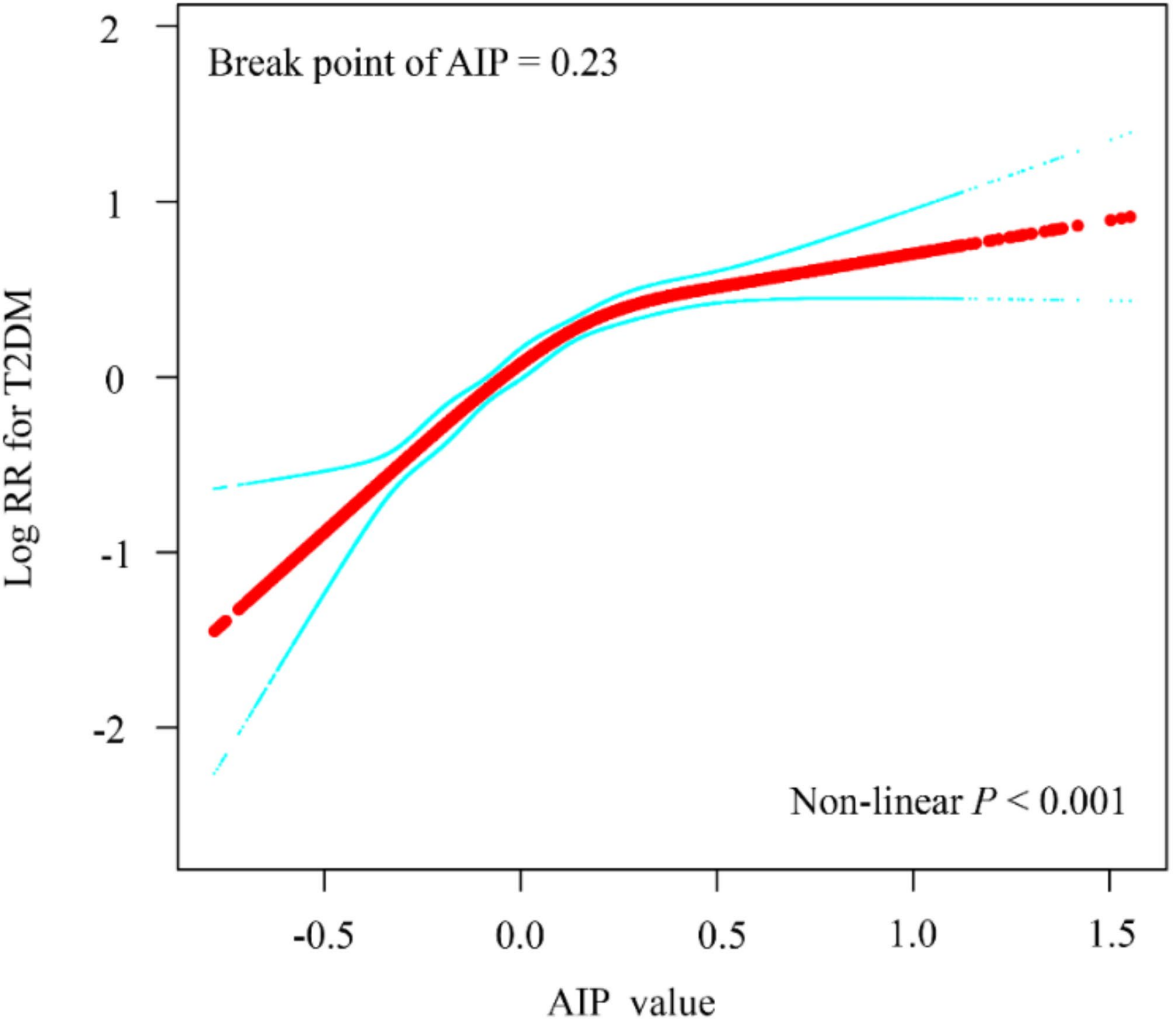
Restricted cubic spline plots for associations of baseline AIP index with T2DM. HRs and 95%CIs for T2DM based on restricted cubic splines for baseline AIP index; HRs and 95%CIs were calculated using Cox proportional hazards models after adjustment for age. HRs, Hazard Ratios; 95% CIs, 95% Confidence Intervals; AIP, plasma atherogenic index; T2DM, type 2 diabetes mellitus

A Cox proportional hazards regression analysis over the follow-up period revealed a significant positive association between the highest quartile (Q4) of AIP and T2DM risk compared with Q1 (HR = 4.61, 95% CI: 4.07–5.23). This relationship remained significant even after adjusting for all potential confounders. In the fully adjusted model, the hazard ratios (HRs) were 1.41 (95% CI: 1.16–1.71) for Q2, 1.75 (95% CI: 1.46–2.11) for Q3, and 2.10 (95% CI: 1.74–2.53) for Q4. As shown in Table 2, the risk of T2DM increased by 41%, 75%, and 110% in Q2, Q3, and Q4, respectively, compared with Q1. Including mean AIP in a sensitivity analysis yielded consistent findings (Supplementary Table S4). Meanwhile, we also examined traditional lipid parameters (TC, LDL-C, TG, and TG/HDL-C) for their predictive value in T2DM. After adjusting confounders, AIP was more strongly associated with T2DM than any of the traditional lipid indicators (Supplementary Table S5). A restricted cubic spline Cox model illustrated the dose–response relationship between AIP and T2DM in this health screening population (Fig. 3). The results indicate a nonlinear positive association between baseline AIP and T2DM risk, with a nonlinear inflection point at an AIP level of 0.23.

**Table 2:** Multivariate regression analysis for T2DM

	Non-adjusted	Model I	Model II
	HR (95% CI) *P*-value	HR (95% CI) *P*-value	HR (95% CI) *P*-value
*AIP quartile*			
Q1	Reference	Reference	Reference
Q2	2.04 (1.77, 2.34) < 0.001	1.65 (1.38, 1.96) < 0.001	1.41 (1.16, 1.71) < 0.001
Q3	3.15 (2.77, 3.59) < 0.001	2.36 (2.00, 2.79) < 0.001	1.75 (1.46, 2.11) < 0.001
Q4	4.61 (4.07, 5.23) < 0.001	3.29 (2.80, 3.87) < 0.001	2.10 (1.74, 2.53) < 0.001
*AIP Trajectory*			
Trajectory 1	Reference	Reference	Reference
Trajectory 2	2.25 (2.00, 2.52) < 0.001	1.96 (1.74, 2.20) < 0.001	1.72 (1.50,1.96) < 0.001
Trajectory 3	2.84 (2.40, 3.35) < 0.001	2.82 (2.38, 3.35) < 0.001	2.50 (2.06, 3.03) < 0.001

Non-adjusted model adjust for: None

Model I adjust for: sex, age, ethnic group, and marriage status

Model II adjust for: sex, age, ethnic group, marriage status, BMI, current drinking, current smoking, hypertension, TP, ALT, AST, BUN, UA, and eGFR

AIP, plasma atherogenic index; HR, Hazard Ratio; 95%CI, 95% Confidence Interval; T2DM, type 2 diabetes mellitus

### Baseline characteristics stratified by AIP trajectory groups

**Fig. 4:**
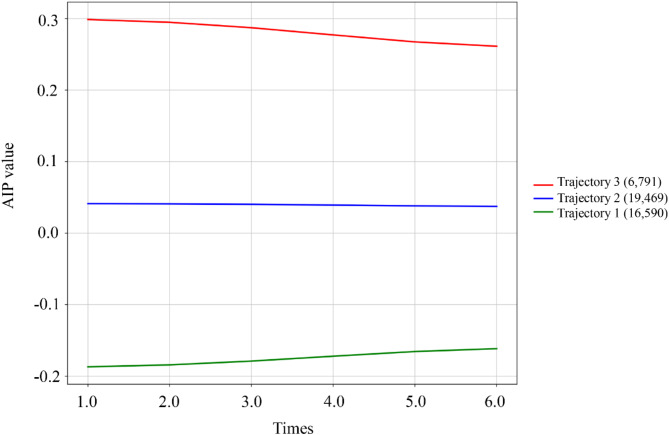
AIP trajectory groups and percentage of participants in each group. Trajectory 1 (*n* = 16,590), low-stable trajectory; Trajectory 2 (*n* = 19,469), moderate-stable trajectory; Trajectory 3 (*n* = 6,791), high-stable trajectory. AIP, atherogenic index of plasma

While baseline AIP provides insight into immediate risk stratification, we further investigated how long-term AIP patterns might influence T2DM development. Using latent class trajectory modeling, we identified three distinct AIP trajectory patterns over the follow-up period. Repeated AIP measurements were categorized into three groups using the LCTM: low-stable group (Trajectory 1, *n* = 16,590), medium-stable group (Trajectory 2, *n* = 19,469), and high-stable group (Trajectory 3, *n* = 6,791) **(**Fig. 4**)**. Table 3 presents the baseline demographic and clinical characteristics for each AIP trajectory group. All variables, except for ethnicity, demonstrated statistically significant differences among the trajectory groups (*P* < 0.001). Specifically, the high-stable group exhibited higher age and BMI, a greater proportion of males, and increased rates of marriage, smoking, alcohol consumption, and hypertension compared to the low-stable AIP group. Furthermore, levels of ALT, AST, BUN, creatinine, UA, FBG, HbA1c, total cholesterol, LDL-C, and TG were significantly elevated, while eGFR and HDL-C levels were notably reduced (all *P* < 0.001). The incidence rates of T2DM for the three groups were 3.10%, 9.16%, and 13.30%, with the high-stable group showing the highest incidence.

**Table 3:** Baseline characterization according to AIP index trajectories

Characteristics	Trajectory 1	Trajectory 2	Trajectory 3	*P* value
N	16,590	19,469	6,791	
AIP	-0.24 (-0.34, -0.14)	0.07 (-0.04, 0.18)	0.40 (0.29, 0.54)	< 0.001
Mean AIP	-0.21 (-0.30, -0.14)	0.08 (0.00, 0.17)	0.41 (0.34, 0.51)	< 0.001
Age, years	41.62 ± 14.90	45.07 ± 14.76	47.80 ± 13.23	< 0.001
Sex, n (%)				< 0.001
Female	11,839 (71.36)	8,169 (41.96)	1,617 (23.81)	
Male	4,751 (28.64)	11,300 (58.04)	5,174 (76.19)	
BMI, kg/m^2^	22.26 ± 2.87	24.80 ± 3.09	26.29 ± 3.07	< 0.001
Ethnic group, n (%)				0.532
Non-han	321 (1.93)	351 (1.80)	117 (4.26)	
Han	16,269 (98.07)	19,118 (98.20)	6,674 (95.74)	
Marriage status, n (%)				< 0.001
Unmarried	3,350 (20.19)	2,155 (11.07)	528 (7.77)	
Married	13,240 (79.81)	17,314 (88.93)	6,263 (92.23)	
Current drinking, n (%)				< 0.001
No	15,887 (95.76)	17,157 (88.12)	5,619 (82.74)	
Yes	703 (4.24)	2,312 (11.88)	1,172 (17.26)	
Current smoking, n (%)				< 0.001
No	15,194 (91.59)	16,894 (86.77)	5,545 (99.18)	
Yes	1,396 (8.41)	2,575 (13.23)	1,246 (18.35)	
Hypertension, n (%)				< 0.001
No	14,547 (87.65)	14,623 (75.11)	4,578 (67.41)	
Yes	2,043 (12.31)	4,846 (24.89)	2,213 (32.59)	
TP, g/L	71.81 ± 4.08	72.22 ± 4.09	72.82 ± 4.14	< 0.001
ALT, U/L	14.50 (11.00,19.60)	19.30 (14.30,27.00)	25.00 (18.08,27.00)	< 0.001
AST, U/L	20.21 ± 9.96	22.05 ± 10.56	24.43 ± 11.78	< 0.001
BUN, mmol/L	4.74 ± 1.27	5.03 ± 1.26	5.12 ± 1.22	< 0.001
Cre, µmol/L	58.04 ± 16.60	63.92 ± 14.67	67.93 ± 14.21	< 0.001
UA, µmol/L	277.84 ± 70.31	330.70 ± 82.43	380.46 ± 89.25	< 0.001
eGFR, mL/min/1.73m^2^	114.10 ± 21.76	108.14 ± 20.28	106.59 ± 19.60	< 0.001
FBG, mmol/L	4.60 ± 0.45	4.79 ± 0.51	4.93 ± 0.54	< 0.001
HbA1c, (%)	5.50 ± 0.38	5.60 ± 0.38	5.62 ± 0.38	< 0.001
TC, mmol/L	4.64 ± 0.85	4.92 ± 0.92	5.09 ± 0.96	< 0.001
LDL-C, mmol/L	2.46 ± 0.66	2.88 ± 0.73	2.82 ± 0.75	< 0.001
TG, mmol/L	0.95 ± 0.34	1.59 ± 0.58	3.14 ± 1.77	< 0.001
HDL-C, mmol/L	1.59 ± 0.28	1.29 ± 0.22	1.07 ± 0.18	< 0.001
T2DM, n (%)				< 0.001
No	16,076(96.90)	17,685(90.84)	5,888(86.70)	
Yes	514(3.10)	1,784(9.16)	903(13.30)	

Trajectory 1, Low gradual trajectory; Trajectory 2, Middle gradual trajectory; Trajectory 3, High gradual trajectory; AIP, plasma atherogenic index; BMI, body mass index; TP, total protein; ALT, alanine aminotransferase; AST, aspartate transaminase; BUN, blood urea nitrogen; Cre, Creatinine; UA, Uric acid; eGFR, estimated glomerular filtration rate; FBG, fasting blood glucose; HbA1c, Glycosylated hemoglobin; TC, total cholesterol; LDL-C, low-density lipoprotein cholesterol; TG, triglycerides; HDL-C, high-density lipoprotein cholesterol; T2DM, type 2 diabetes mellitus. Except for the ALT, AIP, and mean AIP which is expressed as medians (upper and lower quartiles), all other variables are expressed as mean ± standard deviation or counts (percentages)

### Relationship between changes in AIP trajectory and the occurrence of T2DM events

Three models were developed using Trajectory 1 as the reference group to examine the relationship between different AIP trajectories and T2DM, as illustrated in Table 2. The non-adjusted model indicated a positive correlation between AIP levels and T2DM for both Trajectory 2 (HR = 2.25, 95% CI: 2.00, 2.52) and Trajectory 3 (HR = 2.84, 95% CI: 2.40, 3.35). In Model I, which adjusted for certain demographic characteristics, the positive associations persisted, showing increases of 1.96 times for Trajectory 2 and 2.82 times for Trajectory 3. Model II, accounting for all potential confounding factors, confirmed that the positive relationships were robust, with Trajectory 2 (HR = 1.72, 95% CI: 1.50, 1.96) and Trajectory 3 (HR = 2.50, 95% CI: 2.06, 3.03) continuing to show significant correlations with T2DM. Sensitivity analyses further validated that the positive relationships between Trajectories 2 and 3 and T2DM remained consistent (see Supplementary Material, Table S6).

## Discussion

In the field of preventive medicine, identifying a reliable biomarker for T2DM prediction remains a central challenge. Although many lipid parameters and indices have been proposed for assessing diabetes risk, their relative predictive value warrants careful evaluation. By examining both baseline and longitudinal associations between the AIP and T2DM risk in a large-scale health screening population—and comparing AIP to conventional lipid parameters—our study adds valuable evidence to this expanding research area. In this longitudinal study, initiated in January 2018, 42,850 adults undergoing health screenings were followed. After a median follow-up of 47.95 months (until August 2024), we observed a significant link between baseline AIP quartiles, AIP trajectories, and T2DM incidence. Even after adjusting for various risk factors, the impact of AIP on T2DM remained substantial. Our findings underscore the predictive value of AIP levels and their changing patterns for T2DM events in a health screening population. To the best of our knowledge, this is the first large-scale longitudinal cohort study focusing on baseline AIP levels, their trajectories, and T2DM risk in adults undergoing health screenings. These insights offer valuable guidance for T2DM prevention and lipid management in this population.

Previous studies have confirmed a strong correlation between AIP levels and T2DM risk [24–27], further highlighting the predictive value of AIP for T2DM events. In the Kailuan cohort of 52,224 participants, cumulative AIP was independently associated with T2DM risk, unaffected by age, sex, medication use, or blood pressure, underscoring the potential of AIP to identify individuals at high risk [24]. To place our findings in the context of existing literature, we compared the predictive performance of AIP with other established lipid parameters. Our analysis revealed that AIP had a stronger association with T2DM risk compared with traditional indicators (HR = 2.50, 95% CI: 2.06–3.03 in the high-stability trajectory group). When contrasted with conventional lipid metrics, the hazard ratios were 0.93 (95% CI: 0.88–0.98) for TG and 0.94 (95% CI: 0.90–0.98) for TG/HDL-C, whereas TC and LDL-C had no significant associations with T2DM. These comparisons suggest that AIP may offer enhanced predictive utility, potentially due to its combined assessment of TG and HDL-C levels, its log-transformed nature, and its better reflection of lipoprotein particle size.

This study further explored the relationship between AIP trajectories and T2DM risk in a large adult health screening population. Our findings indicate that adults with higher baseline AIP levels had a 1.1-fold greater risk of developing T2DM than those with lower AIP levels. Moreover, once AIP exceeded 0.23, the rate of increased T2DM risk appeared to slow. These results suggest that AIP may be a valuable marker for assessing T2DM risk in the general adult population. A recent Diabetes Cardiovascular Risk Control Action Study involving 10,251 participants identified a critical AIP threshold of 0.34 for T2DM, which is closely aligned with our findings [26]. Another cross-sectional study of 12,060 Chinese adults over 45 years of age found a nonlinear association between AIP and T2DM risk, showing a positive correlation when AIP exceeded − 0.04 [28]. These studies reinforce our conclusions and further validate the predictive value of AIP levels for T2DM events. Furthermore, other research suggests that in overweight and obese populations, an AIP index above − 0.07 significantly increases the risk of T2DM, providing additional support for our findings.

This study employed an LCTM to categorize changes in AIP levels during follow-up into three trajectories. Individuals in the high-stability AIP trajectory group had approximately 1.5 times higher risk of developing T2DM than those in the low-stability group. Previous research has demonstrated that consistently high AIP levels or shifts in AIP between high and low levels are significantly associated with T2DM in middle-aged and older populations [25]. In addition, a recent large-scale longitudinal study revealed that abnormal AIP levels, combined with cumulative inflammation, further raised the incidence of T2DM [24]. Notably, new longitudinal data from a Chinese middle-aged population indicated that varying trajectories of lipid accumulation product (LAP) have sex-specific relationships with T2DM risk. Women transitioning from low to high LAP and both women and men with persistently high LAP exhibited significantly elevated T2DM incidence [29]. These findings support our conclusion that AIP trajectories effectively predict T2DM risk. Moreover, the risk assessment value of long-term AIP trajectories has been applied to investigate associations with hypertension and heart failure [30], the onset of diabetic nephropathy and retinopathy among T2DM patients [31], and cardiovascular disease in the general adult population [32].

T2DM is a complex metabolic disorder associated with various conditions, including cardiovascular disease [33], nonalcoholic fatty liver disease [34], and cancer [35]. Although our findings indicate a strong temporal correlation between AIP and T2DM risk, we acknowledge that this does not imply causation. The relationship between lipid metabolism and glucose homeostasis is intricate and likely bidirectional [36]. Multiple factors—including genetic predisposition, lifestyle habits, and environmental influence may independently or jointly affect AIP levels and T2DM risk [37]. Insulin resistance (IR) is the fundamental pathological mechanism of T2DM, as reduced insulin action in adipose tissue leads to increased free fatty acid (FFA) release, which exacerbates insulin resistance [38, 39]. Elevated FFAs also stimulate hepatic gluconeogenesis, raising blood glucose levels. In an insulin-resistant state, the liver becomes less responsive to insulin, resulting in decreased glycogen synthesis and increased gluconeogenesis, thereby perpetuating hyperglycemia and abnormal lipid metabolism [40]. Abnormal fat accumulation not only releases various inflammatory factors (e.g., tumor necrosis factor-α and interleukin-6) but also disrupts insulin signaling, further worsening insulin resistance [41]. TG, one of the most abundant lipids in circulation, can be toxic at elevated levels, driving the onset and progression of insulin resistance [42]. HDL-C, which contains various lipids and proteins, may influence metabolic disease through its antioxidant and anti-inflammatory properties [43]. AIP integrates TG and HDL-C levels, reflecting their ratio and the size of lipoprotein particles, thereby offering a comprehensive assessment of the pathogenicity and specificity of dyslipidemia. The link between persistently high AIP and increased T2DM risk may be explained by a “vicious cycle,” in which dyslipidemia leads to insulin resistance and elevated insulin levels [44, 45]. Elevated plasma TG competes with glucose for cellular entry, reducing the number and activity of insulin receptors on adipocytes and hindering insulin–receptor binding, ultimately contributing to diabetes [46]. Decreased HDL-C may negatively affect pancreatic β-cell function, diminishing both insulin secretion and sensitivity [25, 46]. Conversely, insulin resistance can also raise TG levels and lower HDL-C [47]. Therefore, prioritizing lipid management is crucial in preventing T2DM in the general healthy population.

Our findings have significant clinical implications for T2DM prevention. First, the results suggest that the AIP can serve as a simple yet effective screening tool for identifying high-risk individuals, relying only on routine lipid measurements commonly obtained in clinical settings. The study identifies a specific AIP threshold (0.23) at which T2DM risk markedly increases, offering clinicians a practical cutoff for immediate risk stratification and early intervention decisions. Second, our trajectory analysis shows that maintaining consistently low AIP levels is crucial for T2DM prevention, suggesting that regular AIP monitoring, especially in individuals with moderate to high levels—may enable earlier interventions. The observed differences in population distribution between baseline AIP groups and trajectory patterns reflect the dynamic nature of AIP changes over time. Some individuals with high baseline AIP may show improvement over time and thus be classified into lower trajectory groups, while others may experience worsening AIP levels despite lower baseline values. This dynamic aspect of AIP adds another dimension to its predictive value for T2DM risk. This dual analytical approach, combining baseline risk assessment and longitudinal monitoring, provides a more comprehensive framework for T2DM prevention. Given that dyslipidemia often precedes T2DM and can be modified through lifestyle changes, this finding is particularly relevant. Third, our results support incorporating AIP monitoring into routine health examinations to improve early risk detection and facilitate timely prevention. The strong correlation between AIP trajectories and T2DM risk (hazard ratio of 2.50 in the high-stability group) provides robust evidence for using AIP as a longitudinal monitoring tool. Future studies should focus on validating these findings in diverse populations and evaluating the cost-effectiveness of AIP-based screening strategies. However, our findings should be interpreted within the context of current clinical practice. Although AIP shows promising potential as a predictive marker, its clinical value is likely maximized when combined with other well-established risk factors and biomarkers. Future research should emphasize developing comprehensive risk assessment models—incorporating AIP among multiple parameters—to enhance T2DM prediction and prevention strategies.

The main strengths of this study include the large volume of longitudinal follow-up data, providing strong statistical support for our analysis. In addition, this is the first study to quantitatively examine the relationship between baseline AIP levels, their trajectories, and T2DM incidence in a health screening population, underscoring the potential of AIP as a predictive tool for T2DM risk. We also performed repeated AIP measurements and tracked T2DM outcomes during follow-up to investigate possible causal links. However, several limitations must be acknowledged. First, although our longitudinal design established a temporal sequence, the observational nature of the study prevents us from drawing definitive causal conclusions. Second, despite adjusting for multiple confounders, unmeasured factors may have introduced residual confounding. Specifically, detailed information on dietary habits, physical activity patterns, and genetic predisposition was not available in our database. These factors could potentially influence both AIP levels and T2DM risk. Third, this study was conducted among health-screened adults in a single center in China, which may introduce potential selection bias through the “healthy user effect.” While our study population showed comparable cardiometabolic risk factors to national survey data, participants undergoing regular health examinations may be more health-conscious and have better access to healthcare, potentially limiting the generalizability of our findings to populations with different socioeconomic backgrounds or healthcare access. Fourth, over approximately six years of follow-up, some participants may not yet have manifested T2DM. Although our comparative analysis of AIP with other lipid parameters is informative, confirmatory research in broader community populations may be needed. In addition, while LCTM provides valuable insights into AIP patterns, we acknowledge that predefined trajectory classes may not capture all individual variations in lipid profiles, and group assignment may introduce some classification uncertainty. Future studies using alternative modeling approaches and incorporating data from multiple screening centers with varying levels of healthcare access and extended follow-up periods might provide additional insights into the complex dynamics of lipid profile changes and further validate our findings.

## Conclusion

In this longitudinal study of health-screened adults, we identified baseline AIP levels, as well as moderate and high stable AIP trajectories, as independent risk factors for T2DM. While our findings provide valuable insights for preventive care settings, future studies should validate these results in more diverse populations, including different healthcare settings and socioeconomic groups.

## Electronic supplementary material

Below is the link to the electronic supplementary material.

Supplementary Material 1

Supplementary Material 2

Supplementary Material 3

Supplementary Material 4

Supplementary Material 5

Supplementary Material 6

Supplementary Material 7

## Data Availability

Contact the first author for all data relating to this study on reasonable request.

## References

[1] Ahmad E, Lim S, Lamptey R, Webb DR, Davies MJ. Type 2 diabetes. Lancet (London England). 2022;400(10365):1803–20. 36332637 10.1016/S0140-6736(22)01655-5

[2] Global, regional, and national burden of diabetes from 1990 to 2021, with projections of prevalence to 2050: a systematic analysis for the Global Burden of Disease Study 2021. Lancet (London England). 2023;402(10397):203–34. 10.1016/S0140-6736(23)01301-6 PMC10364581 37356446

[3] Huang J, Xie Y, Yuan D, Guo L, Qu J, Zhou M. Identification of distinct metabolic characteristics of pneumonia in type 2 diabetes mellitus. Clin Translational Med. 2021;11(2):e303. 10.1002/ctm2.303 PMC7862164 33634967

[4] Shao F, Hu X, Li J, Bai B, Tian L. Lipidomics analysis of impaired glucose tolerance and type 2 diabetes mellitus in overweight or obese elderly adults. Endocr Connect 2023, 12(12). 10.1530/EC-23-0212 PMC10692693 37878774

[5] Yung JHM, Yeung LSN, Ivovic A, Tan YF, Jentz EM, Batchuluun B, Gohil H, Wheeler MB, Joseph JW, Giacca A et al. Prevention of lipotoxicity in pancreatic islets with gammahydroxybutyrate. cells 2022, 11(3). 10.3390/cells11030545 PMC8833960 35159354

[6] Wang Y, Wang XJ, Zhao LM, Pang ZD, She G, Song Z, Cheng X, Du XJ, Deng XL. Oxidative stress induced by palmitic acid modulates K(ca)2.3 channels in vascular endothelium. Exp Cell Res. 2019;383(2):111552. 31415760 10.1016/j.yexcr.2019.111552

[7] Li B, Leung JCK, Chan LYY, Yiu WH, Tang SCW. A global perspective on the crosstalk between saturated fatty acids and toll-like receptor 4 in the etiology of inflammation and insulin resistance. Prog Lipid Res. 2020;77:101020. 31870728 10.1016/j.plipres.2019.101020

[8] Hermans MP, Valensi P. Elevated triglycerides and low high-density lipoprotein cholesterol level as marker of very high risk in type 2 diabetes. Current opinion in endocrinology, diabetes, and obesity 2018, 25(2):118–29. 10.1097/MED.0000000000000398 29493554

[9] Janssen J. Overnutrition, hyperinsulinemia and ectopic fat: it is time for a paradigm shift in the management of type 2 diabetes. Int J Mol Sci 2024, 25(10). 10.3390/ijms25105488 PMC11121669 38791525

[10] Dobiásová M, Frohlich J. The plasma parameter log (TG/HDL-C) as an atherogenic index: correlation with lipoprotein particle size and esterification rate in apob-lipoprotein-depleted plasma (FER(HDL)). Clin Biochem. 2001;34(7):583–8. 11738396 10.1016/s0009-9120(01)00263-6

[11] Zheng X, Zhang X, Han Y, Hu H, Cao C. Nonlinear relationship between atherogenic index of plasma and the risk of prediabetes: a retrospective study based on Chinese adults. Cardiovasc Diabetol. 2023;22(1):205. 37563588 10.1186/s12933-023-01934-0 PMC10416492

[12] Bakillah A, Obeid KK, Al Subaiee M, Soliman AF, Al Arab M, Bashir SF, Al Hussaini A, Al Otaibi A, Mubarak SAS, Iqbal J et al. Association of advanced lipoprotein subpopulation profiles with insulin resistance and inflammation in patients with type 2 diabetes Mellitus. J Clin Med 2023, 12(2). 10.3390/jcm12020487 PMC9864672 36675414

[13] Dobiásová M. AIP–atherogenic index of plasma as a significant predictor of cardiovascular risk: from research to practice. Vnitr Lek. 2006;52(1):64–71. 16526201

[14] Hu Y, Wang X, Luo C, Zheng T, Tian G. Sex difference in the relationship of the atherogenic index of plasma with coronary artery lesions in diabetes: a cross-sectional study. Lipids Health Dis. 2023;22(1):10. 36681828 10.1186/s12944-022-01767-y PMC9862548

[15] Lioy B, Webb RJ, Amirabdollahian F. The Association between the Atherogenic Index of Plasma and cardiometabolic risk factors: a review. Healthc (Basel Switzerland) 2023, 11(7). 10.3390/healthcare11070966 PMC10094587 37046893

[16] Sun Y, Li F, Zhou Y, Liu A, Lin X, Zou Z, Lv X, Zhou J, Li Z, Wu X, et al. Nonlinear association between atherogenic index of plasma and type 2 diabetes mellitus in overweight and obesity patients: evidence from Chinese medical examination data. Cardiovasc Diabetol. 2024;23(1):226. 38951808 10.1186/s12933-024-02330-y PMC11218131

[17] Ma X, Sun Y, Cheng Y, Shen H, Gao F, Qi J, Yang L, Wang Z, Shi D, Liu Y, et al. Prognostic impact of the atherogenic index of plasma in type 2 diabetes mellitus patients with acute coronary syndrome undergoing percutaneous coronary intervention. Lipids Health Dis. 2020;19(1):240. 33198752 10.1186/s12944-020-01418-0 PMC7667811

[18] Yang H, Kuang M, Yang R, Xie G, Sheng G, Zou Y. Evaluation of the role of atherogenic index of plasma in the reversion from Prediabetes to normoglycemia or progression to diabetes: a multi-center retrospective cohort study. Cardiovasc Diabetol. 2024;23(1):17. 38184569 10.1186/s12933-023-02108-8 PMC10771677

[19] Sun X, Zhang J, Nie Q. Inferring latent temporal progression and regulatory networks from cross-sectional transcriptomic data of cancer samples. PLoS Comput Biol. 2021;17(3):e1008379. 33667222 10.1371/journal.pcbi.1008379 PMC7968745

[20] 2. Diagnosis and classification of diabetes: standards of care in diabetes-2024. Diabetes Care. 2024;47(Suppl 1):S20–42. 38078589 10.2337/dc24-S002 PMC10725812

[21] Zhang M, Shi Y, Zhou B, Huang Z, Zhao Z, Li C, Zhang X, Han G, Peng K, Li X, et al. Prevalence, awareness, treatment, and control of hypertension in China, 2004-18: findings from six rounds of a national survey. BMJ (Clinical Res ed). 2023;380:e071952. 10.1136/bmj-2022-071952 PMC10498511 36631148

[22] Levey AS, Coresh J, Greene T, Stevens LA, Zhang YL, Hendriksen S, Kusek JW, Van Lente F. Using standardized serum creatinine values in the modification of diet in renal disease study equation for estimating glomerular filtration rate. Ann Intern Med. 2006;145(4):247–54. 16908915 10.7326/0003-4819-145-4-200608150-00004

[23] Nagin DS, Odgers CL. Group-based trajectory modeling in clinical research. Ann Rev Clin Psychol. 2010;6:109–38. 20192788 10.1146/annurev.clinpsy.121208.131413

[24] Lan Y, Chen G, Wu D, Ding X, Huang Z, Wang X, Balmer L, Li X, Song M, Wang W, et al. Temporal relationship between atherogenic dyslipidemia and inflammation and their joint cumulative effect on type 2 diabetes onset: a longitudinal cohort study. BMC Med. 2023;21(1):31. 36691001 10.1186/s12916-023-02729-6 PMC9870774

[25] Yi Q, Ren Z, Bai G, Zhu S, Li S, Li C, Wu H, Zhu Y, Song P. The longitudinal effect of the atherogenic index of plasma on type 2 diabetes in middle-aged and older Chinese. Acta Diabetol. 2022;59(2):269–79. 34648090 10.1007/s00592-021-01801-y

[26] Fu L, Zhou Y, Sun J, Zhu Z, Xing Z, Zhou S, Wang Y, Tai S. Atherogenic index of plasma is associated with major adverse cardiovascular events in patients with type 2 diabetes mellitus. Cardiovasc Diabetol. 2021;20(1):201. 34610830 10.1186/s12933-021-01393-5 PMC8493717

[27] Yin B, Wu Z, Xia Y, Xiao S, Chen L, Li Y. Non-linear association of atherogenic index of plasma with insulin resistance and type 2 diabetes: a cross-sectional study. Cardiovasc Diabetol. 2023;22(1):157. 37386500 10.1186/s12933-023-01886-5 PMC10311747

[28] Jiang L, Li L, Xu Z, Tang Y, Zhai Y, Fu X, Liu D, Wu Q. Non-linear associations of atherogenic index of plasma with prediabetes and type 2 diabetes mellitus among Chinese adults aged 45 years and above: a cross-sectional study from CHARLS. Front Endocrinol. 2024;15:1360874. 10.3389/fendo.2024.1360874 PMC11018972 38628590

[29] Yu J, Yi Q, Hou L, Chen G, Shen Y, Song Y, Zhu Y, Song P. Transition of lipid accumulation product status and the risk of type 2 diabetes mellitus in middle-aged and older Chinese: a National Cohort Study. Front Endocrinol. 2021;12:770200. 10.3389/fendo.2021.770200 PMC8660859 34899605

[30] Zheng H, Huang Z, Wu K, Wu W, Wang X, Fu P, Wang Y, Chen Z, Cai Z, Cai Z, et al. Association between the atherogenic index of plasma trajectory and risk of heart failure among hypertensive patients: a prospective cohort study. Cardiovasc Diabetol. 2024;23(1):301. 39152490 10.1186/s12933-024-02375-z PMC11330004

[31] Zhang J, Liu C, Peng Y, Fang Q, Wei X, Zhang C, Sun L, Hu Z, Hong J, Gu W, et al. Impact of baseline and trajectory of the atherogenic index of plasma on incident diabetic kidney disease and retinopathy in participants with type 2 diabetes: a longitudinal cohort study. Lipids Health Dis. 2024;23(1):11. 38212770 10.1186/s12944-024-02003-5 PMC10782533

[32] Chun DW, Lee YJ, Lee JH, Lee JW. Longitudinal trajectories of atherogenic index of plasma and risks of cardiovascular diseases: results from the Korean genome and epidemiology study. Thromb J. 2023;21(1):99. 37723571 10.1186/s12959-023-00542-y PMC10506251

[33] Davies MJ, Aroda VR, Collins BS, Gabbay RA, Green J, Maruthur NM, Rosas SE, Del Prato S, Mathieu C, Mingrone G, et al. Management of hyperglycemia in type 2 diabetes, 2022. A Consensus Report by the American Diabetes Association (ADA) and the European Association for the Study of Diabetes (EASD). Diabetes Care. 2022;45(11):2753–86. 36148880 10.2337/dci22-0034 PMC10008140

[34] Tanase DM, Gosav EM, Costea CF, Ciocoiu M, Lacatusu CM, Maranduca MA, Ouatu A, Floria M. The Intricate Relationship between Type 2 Diabetes Mellitus (T2DM), Insulin Resistance (IR), and Nonalcoholic Fatty Liver Disease (NAFLD). J Diabetes Res 2020, 2020:3920196. 10.1155/2020/3920196 PMC7424491 32832560

[35] Zhang YY, Li YJ, Xue CD, Li S, Gao ZN, Qin KR. Effects of T2DM on cancer progression: pivotal precipitating factors and underlying mechanisms. Front Endocrinol. 2024;15:1396022. 10.3389/fendo.2024.1396022 PMC11405243 39290325

[36] Jaishy B, Abel ED. Lipids, lysosomes, and autophagy. J Lipid Res. 2016;57(9):1619–35. 27330054 10.1194/jlr.R067520 PMC5003162

[37] Li R, Cai M, Qian ZM, Wang X, Zhang Z, Wang C, Wang Y, Arnold LD, Howard SW, Li H, et al. Ambient air pollution, lifestyle, and genetic predisposition associated with type 2 diabetes: findings from a national prospective cohort study. Sci Total Environ. 2022;849:157838. 35934032 10.1016/j.scitotenv.2022.157838

[38] Hotamisligil GS. Inflammation and metabolic disorders. Nature. 2006;444(7121):860–7. 17167474 10.1038/nature05485

[39] Chen L, Chen XW, Huang X, Song BL, Wang Y, Wang Y. Regulation of glucose and lipid metabolism in health and disease. Sci China Life Sci. 2019;62(11):1420–58. 31686320 10.1007/s11427-019-1563-3

[40] Ndumele CE, Pradhan AD, Ridker PM. Interrelationships between inflammation, C-reactive protein, and insulin resistance. J Cardiometab Syndr. 2006;1(3):190–6. 17679826 10.1111/j.1559-4564.2006.05538.x

[41] Rohm TV, Meier DT, Olefsky JM, Donath MY. Inflammation in obesity, diabetes, and related disorders. Immunity. 2022;55(1):31–55. 35021057 10.1016/j.immuni.2021.12.013 PMC8773457

[42] Manell H, Kristinsson H, Kullberg J, Ubhayasekera SJK, Mörwald K, Staaf J, Cadamuro J, Zsoldos F, Göpel S, Sargsyan E, et al. Hyperglucagonemia in youth is associated with high plasma free fatty acids, visceral adiposity, and impaired glucose tolerance. Pediatr Diabetes. 2019;20(7):880–91. 31271247 10.1111/pedi.12890

[43] Di Bartolo BA, Cartland SP, Genner S, Manuneedhi Cholan P, Vellozzi M, Rye KA, Kavurma MM. HDL Improves Cholesterol and Glucose Homeostasis and Reduces Atherosclerosis in Diabetes-Associated Atherosclerosis. J Diabetes Res 2021;6668506. 10.1155/2021/6668506 PMC8163542 34095317

[44] Steiner G, Vranic M. Hyperinsulinemia and hypertriglyceridemia, a vicious cycle with atherogenic potential. Int J Obes. 1982;6(Suppl 1):117–24. 6749716

[45] Li N, Fu J, Koonen DP, Kuivenhoven JA, Snieder H, Hofker MH. Are hypertriglyceridemia and low HDL causal factors in the development of insulin resistance? Atherosclerosis. 2014;233(1):130–8. 10.1016/j.atherosclerosis.2013.12.013 24529133

[46] Goodpaster BH, Kelley DE. Skeletal muscle triglyceride: marker or mediator of obesity-induced insulin resistance in type 2 diabetes mellitus? Curr Diab Rep. 2002;2(3):216–22. 12643176 10.1007/s11892-002-0086-2

[47] Hsu H, Hsu P, Cheng MH, Ito Y, Kanda E, Schaefer EJ, Ai M. Lipoprotein subfractions and glucose homeostasis in prediabetes and diabetes in Taiwan. J Atheroscler Thromb. 2019;26(10):890–914. 30726792 10.5551/jat.48330 PMC6800394

