# Supplementary material for: Baseline atherogenic index of plasma and its trajectory predict onset of type 2 diabetes in a health screened adult population: a large longitudinal study
Source: Cardiovasc Diabetol. 2025 Feb 7;24:57. doi: 10.1186/s12933-025-02619-6 (PMC11806864; doi:10.1186/s12933-025-02619-6)
Supplement: Supplementary file 1 — Supplementary Material 1 [file 12933_2025_2619_MOESM1_ESM.docx]

**Table S1.** Baseline characteristics and T2DM incidence in total participants

| **Characteristics** | Total |
| --- | --- |
| **N** | 42,850 |
| **AIP** | -0.01 (-0.02, 0.19) |
| **Mean AIP** | 0.01 (-0.17, 0.19) |
| **Age, years** | 44.87 ± 14.81 |
| **Sex, n (%)** |  |
| Female | 21,122 (49.29) |
| Male | 21,728 (50.71) |
| **BMI, kg/m^2^** | 24.06 ± 3.38 |
| **Ethnic group, n (%)** |  |
| Non-han | 769 (1.79) |
| Han | 42,081 (98.21) |
| **Marriage status, n (%)** |  |
| Unmarried | 5,074 (11.84) |
| Married | 37,776 (88.16) |
| **Current drinking, n (%)** |  |
| No | 38,663 (90.23) |
| Yes | 4,187 (9.77) |
| **Current smoking, n (%)** |  |
| No | 37,633 (87.82) |
| Yes | 5,217 (12.18) |
| **Hypertension, n (%)** |  |
| No | 33,748 (78.76) |
| Yes | 9,102 (21.24) |
| **TP, g/L** | 72.17 ± 4.10 |
| **ALT, U/L** | 18.00 (13.00, 25.90) |
| **AST, U/L** | 27.12 ± 10.64 |
| **BUN, mmol/L** | 4.93 ± 1.26 |
| **Cre, μmol/L** | 62.40 ± 15.68 |
| **UA, μmol/L** | 318.98 ± 86.89 |
| **eGFR, mL/min/1.73m^2^** | 110.04 ± 20.94 |
| **FBG, mmol/L** | 4.74 ± 0.50 |
| **HbA1c, (%)** | 5.57 ± 0.38 |
| **TC, mmol/L** | 4.84 ± 0.92 |
| **LDL-C, mmol/L** | 2.71 ± 0.74 |
| **TG, mmol/L** | 1.60 ± 1.11 |
| **HDL-C, mmol/L** | 1.37 ± 0.30 |
| **Time, month** | 47.95 ± 14.68 |
| **T2DM, n (%)** |  |
| No | 39,649 (92.53) |
| Yes | 3,201(7.47) |

AIP, plasma atherogenic index; BMI, body mass index; TP, total protein; ALT, alanine aminotransferase; AST, aspartate transaminase; BUN, blood urea nitrogen; Cre, Creatinine; UA, Uric acid; eGFR, estimated glomerular filtration rate; FBG, fasting blood glucose; HbA1c, Glycosylated hemoglobin; TC, total cholesterol; LDL-C, low-density lipoprotein cholesterol; TG, triglycerides; HDL-C, high-density lipoprotein cholesterol; T2DM, type 2 diabetes mellitus. Except for the AST, AIP, and mean AIP which is expressed as medians (upper and lower quartiles), all other variables are expressed as mean ± standard deviation or counts (percentages).
