# Supplementary material for: Baseline atherogenic index of plasma and its trajectory predict onset of type 2 diabetes in a health screened adult population: a large longitudinal study
Source: Cardiovasc Diabetol. 2025 Feb 7;24:57. doi: 10.1186/s12933-025-02619-6 (PMC11806864; doi:10.1186/s12933-025-02619-6)
Supplement: Supplementary file 2 — Supplementary Material 2 [file 12933_2025_2619_MOESM2_ESM.docx]

**Table S5** Baseline characteristics according to the baseline AIP tertiles

| **Characteristics** | **T1 (-0.98, -0.13)** | | **T2 (-0.13, 0.12)** | **T3 (0.12, 1.55)** | ***P*-value** |
| --- | --- | --- | --- | --- | --- |
| **N** | 14,283 | | 14,280 | 14,287 | <0.001 |
| **AIP** | -0.27 (-0.35, -0.19) | | -0.01 (-0.07, 0.05) | 0.29 (0.19, 0.42) | <0.001 |
| **Mean AIP** | -0.23 (-0.31, -0.14) | | 0.00 (-0.08, 0.09) | 0.26 (0.15, 0.39) | <0.001 |
| **Age, years** | 42.10 ± 14.70 | | 47.16 ± 14.81 | 47.98 ± 13.66 | <0.001 |
| **Sex, n (%)** |  | |  |  | <0.001 |
| Female | 10,023 (70.17) | | 6,743 (47.22) | 4,356 (30.49) |  |
| Male | 4260 (29.83) | | 7,537 (52.78) | 9, 931 (69.51) |  |
| **BMI, kg/m^2^** | 22.25 ± 2.83 | | 24.29 ± 2.99 | 26.02 ± 3.05 | <0.001 |
| **Ethnic group, n (%)** |  | |  |  | 0.072 |
| Non-han | 291 (3.27) | | 230 (2.68) | 248 (2.94) |  |
| Han | 8,617 (96.73) | | 8, 353 (97.32) | 8,180 (97.06) |  |
| **Marriage status, n (%)** |  | |  |  | <0.001 |
| Unmarried | 2,783 (19.73) | | 1,416 (10.12) | 875 (6.29) |  |
| Married | 11,319 (80.27) | | 12,583 (89.88) | 13,045 (93.71) |  |
| **Current drinking, n (%)** |  | |  |  | <0.001 |
| No | 13,795(96.33) | | 12,854 (90.01) | 12,050 (84.34) |  |
| Yes | 524 (3.67) | | 1,426 (9.99) | 2,237 (15.66) |  |
| **Current smoking, n (%)** |  | |  |  | <0.001 |
| No | 13,670 (95.71) | | 12,643 (88.54) | 11,657 (81.59) |  |
| Yes | 613 (4.29) | | 1,637 (11.46) | 2,630 (18.41) |  |
| **Hypertension, n (%)** |  | |  |  | <0.001 |
| No | 11,071 (86.08) | | 10,064 (76.55) | 8,983 (68.00) |  |
| Yes | 1,791 (13.92) | | 3,083 (23.45) | 4,228 (32.00) |  |
| **TP, g/L** | 71.83 ± 4.00 | | 72.03 ± 4.12 | 72.59 ± 4.15 | <0.001 |
| **ALT, U/L** |  | |  |  | <0.001 |
| **AST, U/L** | 20.25 ± 9.78 | | 21.57 ± 8.86 | 23.71 ± 12.36 | <0.001 |
| **BUN, mmol/L** | 4.81 ± 1.28 | | 4.95 ± 1.28 | 5.06 ± 1.22 | <0.001 |
| **Cre, μmol/L** | 58.24 ± 13.83 | | 62.92 ± 17.69 | 66.46 ± 14.40 | <0.001 |
| **UA, μmol/L** | 278.00 ± 70.31 | | 318.24 ± 80.21 | 361.95 ± 88.09 | <0.001 |
| **eGFR, mL/min/1.73m^2^** | 113.56 ± 21.22 | | 109.02 ± 20.94 | 106.46 ± 19.87 | <0.001 |
| **FBG, mmol/L** | 4.60 ± 0.44 | | 4.74 ± 0.49 | 4.88 ± 0.54 | <0.001 |
| **HbA1c, (%)** | 5.49 ± 0.38 | | 5.58 ± 0.37 | 5.63 ± 0.38 | <0.001 |
| **TC, mmol/L** | 4.61 ± 0.84 | | 4.85 ± 0.91 | 5.07 ± 0.94 | <0.001 |
| **LDL-C, mmol/L** | 2.44 ± 0.66 | | 2.81 ± 0.73 | 2.88 ± 0.74 | <0.001 |
| **TG, mmol/L** | 0.85 ± 0.20 | 1.34 ± 0.27 | | 2.61 ± 1.39 | <0.001 |
| **HDL-C, mmol/L** | 1.61 ± 0.28 | 1.35 ± 0.22 | | 1.13 ± 0.20 | <0.001 |
| **T2DM, n (%)** |  |  | |  | <0.001 |
| No | 13,797 (96.60) | 13,296 (93.11) | | 12,650 (88.54) |  |
| Yes | 486 (3.40) | 984 (6.89) | | 1,637 (11.46) |  |

AIP, plasma atherogenic index; BMI, body mass index; TP, total protein; ALT, alanine aminotransferase; AST, aspartate transaminase; BUN, blood urea nitrogen; Cre, Creatinine; UA, Uric acid; eGFR, estimated glomerular filtration rate; FBG, fasting blood glucose; HbA1c, Glycosylated hemoglobin; TC, total cholesterol; LDL-C, low-density lipoprotein cholesterol; TG, triglycerides; HDL-C, high-density lipoprotein cholesterol; T2DM, type 2 diabetes mellitus. Except for the AIP, mean AIP, and ALT which is expressed as medians (upper and lower quartiles), all other variables are expressed as mean ± standard deviation or counts (percentages).
