# Supplementary material for: Baseline atherogenic index of plasma and its trajectory predict onset of type 2 diabetes in a health screened adult population: a large longitudinal study
Source: Cardiovasc Diabetol. 2025 Feb 7;24:57. doi: 10.1186/s12933-025-02619-6 (PMC11806864; doi:10.1186/s12933-025-02619-6)
Supplement: Supplementary file 3 — Supplementary Material 3 [file 12933_2025_2619_MOESM3_ESM.docx]

**Table S6** Comparison of T2DM incidence rates between baseline AIP tertiles and trajectory groups

| **Classification Method** | **Groups** | **N (%)** | **T2DM** | **HR (95% CI)** | ***P*** |
| --- | --- | --- | --- | --- | --- |
| **Baseline AIP Tertiles** | T1 | 14,283 | 486 (3.40) | Reference |  |
|  | T2 | 14,280 | 984 (6.89) | 1.30 (1.21,1.51) | <0.001 |
|  | T3 | 14,287 | 1,637 (11.46) | 1.72 (1.48, 1.99) | <0.001 |
| **Trajectory Groups** | Trajectory 1 | 16,590 | 514 (3.10) | Reference |  |
|  | Trajectory 2 | 19,469 | 1,784 (9.16) | 1.72 (1.50,1.96) | <0.001 |
|  | Trajectory 3 | 6,791 | 903 (13.30) | 2.50 (2.06, 3.03) | <0.001 |

Adjust for: sex, age, ethnic group, marriage status, BMI, current drinking, current smoking, hypertension, TP, ALT, AST, BUN, UA, and eGFR.

AIP, plasma atherogenic index; HR, Hazard Ratio; 95%CI, 95% Confidence Interval; T2DM, type 2 diabetes mellitus.
