# Supplementary figures and images for: Baseline atherogenic index of plasma and its trajectory predict onset of type 2 diabetes in a health screened adult population: a large longitudinal study
Source: Cardiovasc Diabetol. 2025 Feb 7;24:57. doi: 10.1186/s12933-025-02619-6 (PMC11806864; doi:10.1186/s12933-025-02619-6)

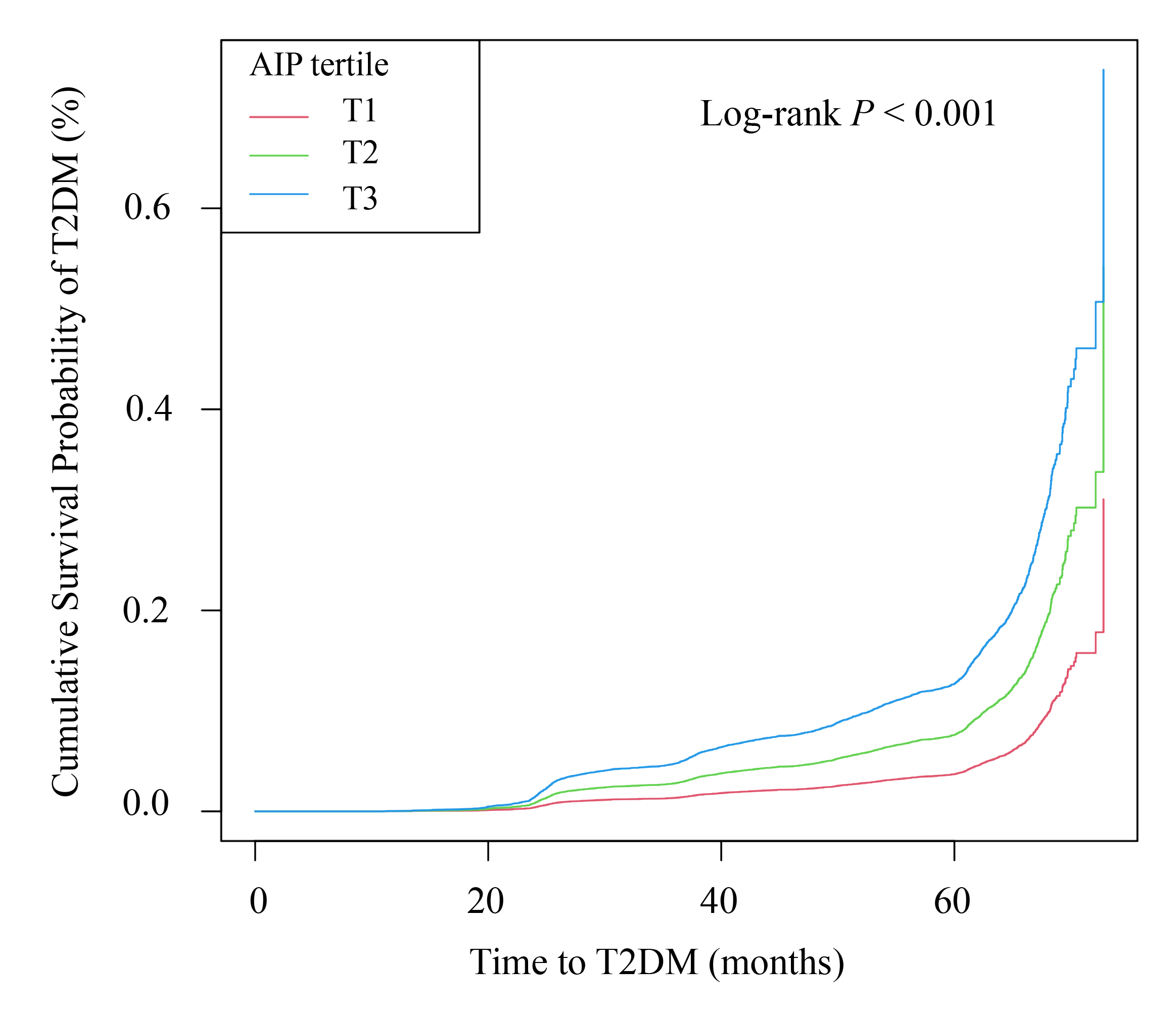

Supplement: Supplementary file 4 — Supplementary Material 4 [file 12933_2025_2619_MOESM4_ESM.tif]
