# Supplementary material for: Baseline atherogenic index of plasma and its trajectory predict onset of type 2 diabetes in a health screened adult population: a large longitudinal study
Source: Cardiovasc Diabetol. 2025 Feb 7;24:57. doi: 10.1186/s12933-025-02619-6 (PMC11806864; doi:10.1186/s12933-025-02619-6)
Supplement: Supplementary file 5 — Supplementary Material 5 [file 12933_2025_2619_MOESM5_ESM.docx]

**Table S2.** Sensitivity analysis of the association between the baseline AIP and T2DM

|  | HR | 95%CI | *P* |
| --- | --- | --- | --- |
| T2DM |  |  |  |
| Q1 | Reference |  |  |
| Q2 | 1.37 | 1.12, 1.67 | 0.002 |
| Q3 | 1.46 | 1.30, 1.98 | <0.001 |
| Q4 | 1.70 | 1.13, 1.89 | 0.004 |

Model adjust for: sex, age, ethnic group, marriage status, BMI, current drinking, current smoking, hypertension, TP, ALT, AST, BUN, UA, eGFR and mean AIP. HR, Hazard Ratio; 95%CI, 95% Confidence Interval; T2DM, type 2 diabetes mellitus.
