# Supplementary material for: Baseline atherogenic index of plasma and its trajectory predict onset of type 2 diabetes in a health screened adult population: a large longitudinal study
Source: Cardiovasc Diabetol. 2025 Feb 7;24:57. doi: 10.1186/s12933-025-02619-6 (PMC11806864; doi:10.1186/s12933-025-02619-6)
Supplement: Supplementary file 6 — Supplementary Material 6 [file 12933_2025_2619_MOESM6_ESM.docx]

**Table S3.** Multivariate regression analysis for T2DM

|  | **Non-adjusted** | **Model I** | **Model II** |
| --- | --- | --- | --- |
|  | **HR (95% CI) *P-*value** | **HR (95% CI) *P-*value** | **HR (95% CI) *P-*value** |
| **TC** | 1.18 (1.14, 1.22) <0.001 | 1.08 (1.03, 1.14) 0.002 | 0.97 (0.92, 1.02) 0.191 |
| **TC quartile** |  |  |  |
| **Q1** | Reference | Reference | Reference |
| **Q2** | 0.92 (0.83, 1.03) 0.147 | 0.97 (0.83, 1.12) 0.633 | 0.89 (0.77, 1.04) 0.134 |
| **Q3** | 1.14 (1.03, 1.27) 0.0109 | 1.08 (0.94, 1.24) 0.265 | 0.92 (0.80, 1.06) 0.243 |
| **Q4** | 1.43 (1.30, 1.58) <0.001 | 1.13 (0.99, 1.29) 0.073 | 0.87 (0.76, 1.00) 0.045 |
| **LDL-C** | 1.16 (1.11, 1.22) <0.001 | 1.01 (0.95, 1.07) 0.772 | 0.95 (0.90, 1.02) 0.155 |
| **LDL-C quartile** |  |  |  |
| **Q1** | Reference | Reference | Reference |
| **Q2** | 0.91 (0.81, 1.01) 0.067 | 0.88 (0.76, 1.01) 0.073 | 0.84 (0.72, 0.97) 0.017 |
| **Q3** | 1.10 (0.99, 1.21) 0.073 | 0.97 (0.84, 1.11) 0.649 | 0.93 (0.81, 1.07) 0.313 |
| **Q4** | 1.30 (1.18, 1.43) <0.001 | 1.00 (0.87, 1.14) 0.944 | 0.87 (0.76, 1.00) 0.047 |
| **TG** | 1.19 (1.18, 1.21) <0.001 | 1.15 (1.12, 1.18) <0.001 | 0.93 (0.88, 0.98) 0.004 |
| **TG quartile** |  |  |  |
| **Q1** | Reference | Reference | Reference |
| **Q2** | 1.81 (1.59, 2.08) <0.001 | 1.29 (1.08, 1.55) 0.005 | 1.07 (0.88, 1.29) 0.505 |
| **Q3** | 2.70 (2.38, 3.06) <0.001 | 1.70 (1.43, 2.02) <0.001 | 1.14 (0.94, 1.39) 0.173 |
| **Q4** | 3.89 (3.44, 4.39) <0.001 | 2.17 (1.84, 2.57) <0.001 | 1.06 (0.84, 1.33) 0.625 |
| **TG/HDL-C** | 1.12 (1.11, 1.13) <0.001 | 1.10 (1.08, 1.12) <0.001 | 0.94 (0.90, 0.98) 0.003 |
| **TG/HDL-C quartile** |  |  |  |
| **Q1** | Reference | Reference | Reference |
| **Q2** | 2.02 (1.76, 2.32) <0.001 | 1.51 (1.25, 1.83) <0.001 | 1.13 (1.09, 1.62) 0.006 |
| **Q3** | 2.99 (2.63, 3.41) <0.001 | 2.00 (1.67, 2.40) <0.001 | 1.23 (1.16, 1.56) <0.001 |
| **Q4** | 4.31 (3.80, 4.88) <0.001 | 2.52 (2.11, 3.01) <0.001 | 1.08 (1.00, 1.48) 0.013 |

Non-adjusted model adjust for: None.

Model I adjust for: sex, age, ethnic group, and marriage status.

Model II adjust for: sex, age, ethnic group, marriage status, BMI, current drinking, current smoking, hypertension, TP, ALT, AST, BUN, UA, and eGFR.

AIP, plasma atherogenic index; HR, Hazard Ratio; 95%CI, 95% Confidence Interval; T2DM, type 2 diabetes mellitus.
