# Supplementary material for: Baseline atherogenic index of plasma and its trajectory predict onset of type 2 diabetes in a health screened adult population: a large longitudinal study
Source: Cardiovasc Diabetol. 2025 Feb 7;24:57. doi: 10.1186/s12933-025-02619-6 (PMC11806864; doi:10.1186/s12933-025-02619-6)
Supplement: Supplementary file 7 — Supplementary Material 7 [file 12933_2025_2619_MOESM7_ESM.docx]

**Table S4.** Sensitivity analysis of the association between the trajectories of the AIP and T2DM

|  | HR | 95%CI | *P* |
| --- | --- | --- | --- |
| T2DM |  |  |  |
| Trajectory1 | Reference |  |  |
| Trajectory2 | 1.26 | 1.08, 1.47 | 0.003 |
| Trajectory3 | 1.30 | 1.01, 1.67 | 0.046 |

Model adjust for: sex, age, ethnic group, marriage status, BMI, current drinking, current smoking, hypertension, TP, ALT, AST, BUN, UA, eGFR and mean AIP. Trajectory 1, High gradual trajectory; Trajectory 2, Middle gradual trajectory; Trajectory 3, Low gradual trajectory; T2DM, type 2 diabetes mellitus.
